# Responses to Traumatic Brain Injury Screening Questions and Suicide Attempts among Those Seeking Veterans Health Administration Mental Health Services

**DOI:** 10.3389/fpsyt.2016.00059

**Published:** 2016-04-19

**Authors:** Alexandra L. Schneider, Trisha A. Hostetter, Beeta Y. Homaifar, Jeri E. Forster, Jennifer H. Olson-Madden, Bridget B. Matarazzo, Joe Huggins, Lisa A. Brenner

**Affiliations:** ^1^Rocky Mountain Mental Illness Research, Education and Clinical Center, U.S. Department of Veterans Affairs, Denver, CO, USA; ^2^Department of Psychiatry, University of Colorado School of Medicine Anschutz Medical Campus, Aurora, CO, USA; ^3^Department of Physical Medicine and Rehabilitation, University of Colorado School of Medicine Anschutz Medical Campus, Aurora, CO, USA; ^4^Department of Biostatistics and Informatics, Colorado School of Public Health, Aurora, CO, USA; ^5^Department of Neurology, University of Colorado School of Medicine Anschutz Medical Campus, Aurora, CO, USA

**Keywords:** traumatic brain injury, suicide, Veteran, psychometrics, predictive validity, screening

## Abstract

**Background:**

Psychometrically sound screening tools available to aid in the identification of lifetime history of traumatic brain injury (TBI) are limited. As such, the Traumatic Brain Injury-4 (TBI-4) was developed and implemented in a Veterans Health Administration (VHA) mental health clinic. To provide information regarding both the predictive validity and clinical utility of the TBI-4, the relationship between screening results and future suicide attempts was evaluated.

**Objective:**

The aim of this study was to determine whether a positive screen on the TBI-4 was associated with increased risk for suicide attempt within 1-year post screening.

**Methods:**

The TBI-4 was administered to 1,097 Veterans at the time of mental health intake. Follow-up data regarding suicide attempts for the year post-mental health intake were obtained from suicide behavior reports (SBRs) in Veteran electronic medical records (EMRs). Fisher’s exact tests were used to determine the proportion of suicide attempts by TBI-4 status.

**Results:**

In the year post TBI-4 screening, significantly more Veterans who screened positive had a documented suicide attempt as compared to those who screened negative (*p* = 0.003).

**Conclusion:**

Those with a positive TBI screen at mental health intake had a higher proportion of SBRs than those who screened negative for TBI. Findings provided further psychometric support for the TBI-4. Moreover, results suggest the inclusion of this screen could prove to be helpful in identifying those who may be at risk for future suicide attempt within 1-year post screening.

## Introduction

Traumatic brain injury (TBI) is a frequently noted health condition among individuals seeking Veteran’s Health Administration (VHA) services. For example, work by Brenner and colleagues (1) suggested that 45% Veterans seeking services at one mental health clinic had a probable history of lifetime TBI. As such, the Department of Veterans Affairs (VA) has made TBI screening, diagnosis, and treatment a priority (2). Although recent attention has been focused on developing a screening tool for deployment-related TBI for Service Members returning from the conflicts in Iraq and Afghanistan (3), there remains a lack of psychometrically sound screening tools for lifetime history of TBI that could be used across Veteran cohorts.

The Traumatic Brain Injury-4 (TBI-4) was developed to address this need and was implemented as part of the mental health intake process in a Veterans Affairs Medical Center Mental Health Clinic (1). It is composed of the following four questions: (1) Have you ever been hospitalized or treated in an emergency room following a head or neck injury?; (2) Have you ever been knocked out or unconscious following an accident or injury?; (3) Have you ever injured your head or neck in a car accident or from some other moving vehicle accident?; and (4) Have you ever injured your head or neck in a fight or fall? The second question, which specifically asks about a loss of consciousness as a result of an accident or injury, contains elements necessary to meet TBI diagnostic criteria (injury event with an associated alteration in consciousness), whereas the other three questions (1, 3, 4) solicit information about risky situations and behaviors that are commonly associated with sustaining a TBI (4). As part of a study, evaluating the prevalence of TBI among Veterans seeking VA mental health services (1) patients’ responses to the TBI-4, administered as part of their mental health intake, were compared to their results from the Ohio State University TBI-ID (OSU TBI-ID), a structured clinical interview for identifying lifetime history of TBI (5). The prevalence of probable lifetime history of TBI, defined as a positive response to Q2 of the TBI-4, within the study population was 45% (95% CI, 42–47) (1), which was significantly higher (*p* < 0.0001) than was reported in previous work examining a 1-item TBI screener in individuals seeking substance abuse treatment (31.7%) (6). Using the OSU TBI-ID (5) as the criterion standard for establishing probable lifetime history of TBI and a positive response to Question 2 as the criterion for a positive screen, the sensitivity and specificity of the TBI-4 were 0.58 and 0.77, respectively (1).

It should also be noted that the practice of screening for TBI, particularly among Operation Enduring Freedom/Operation Iraqi Freedom (OEF/OIF) Veterans is not without some controversy. According to Vanderploeg and Belanger (7), screening procedures should be implemented “to identify a disease, condition, or risk factor early, thus enabling earlier intervention and management in the hope of reducing mortality and suffering” (p. 212). Regarding history of mild TBI, they suggested that these criteria were not met (e.g., mild TBI is not a progressive condition). In response, Bahraini and Brenner (8) suggested that the costs versus benefits of TBI screening should be evaluated empirically and predicated, at least in part, on whether screening can help identify risk factors for other health conditions. In specific, Bahraini and Brenner (8) suggested that TBI screening may inform prevention strategies aimed at reducing risky behaviors (e.g., suicide attempts).

Moreover, evaluating whether a screening tool is predictive of outcomes often associated with a condition of interest can provide additional psychometric support. For example, Olson-Madden and colleagues (9) found that individuals with a positive screen on the TBI-4 (defined as a positive response to Question 2) had more psychiatric hospital stays than those with a negative screen. The authors suggested that this finding provided support for implementation of this screening tool in VHA mental health settings.

Bahraini et al. (10) performed a systematic review of studies related to TBI and suicide published since the beginning of 2007. Evidence from the review provided support for the association between TBI and elevated suicide risk. Studies included in the review found increased risk for death by suicide following TBI in civilian populations with moderate risk of bias (11) and Veteran populations (12) with low risk of bias. A case–control study examining all suicide deaths among those serving in the United States military with moderate risk of bias did not show a significantly higher rate of TBI among those who died by suicide (13). Two studies examining whether Veterans with comorbid PTSD and mild TBI had an increased likelihood of suicidal behaviors as compared to Veterans with PTSD alone reported non-significant results and had high risk of bias (14, 15). Another study found a significantly higher rate of suicide ideation among patients with TBI as compared to healthy controls with moderate risk of bias (16). Overall, there is evidence suggesting that those with a history of TBI are at risk for suicidal behavior (10, 17). As such, it was hypothesized that Veterans who screened positive to a one-question screen for TBI (Question 2 of the TBI-4), in the context of the 4-question TBI screener, would have more suicide attempts during a 1-year follow-up period then those with a negative screen.

## Materials and Methods

### Cohort

The study sample includes 1,097 Veterans who were given the TBI-4 by a mental health clinician during the standard VA Eastern Colorado Health Care System (ECHCS) mental health intake, between December 2006 and February 2010, and did not seek care at any other VA facility during the 1-year follow-up period. Demographic information was obtained from the Veterans Health Administration (VHA) Corporate Data Warehouse (CDW), which is a national repository of data from VHA clinical and administrative systems. The study was approved by the Colorado Multiple Institutional Review Board (COMIRB), as well as the Eastern Colorado Healthcare System Research and Development Service.

### Outcome Measures

#### Traumatic Brain Injury-4

The TBI-4 was used to identify individuals who had probable lifetime history of TBI. The TBI-4 is a four-question screener used to identify those with a probable lifetime history of TBI. The questions are designed with wording chosen to minimize stigma (i.e., “head” versus “brain”) (6). The screen is also consistent with recommendations from the Center for Disease Control and Prevention (18) concerning screening for acute injuries. Individuals who answered “Yes” to the second question on the TBI-4, “Have you ever been knocked out or unconscious following an accident or injury?” were considered to have screened positive. TBI-4 data were collected from local electronic medical records (EMRs).

#### Suicide Attempts

It is VHA policy that all facilities identify and track patients at high risk for suicide. A suicide behavior report (SBR) is the mandatory documentation completed by VA clinical staff members within a patient’s EMR when they become aware of “serious suicidal ideation” or suicidal behaviors (i.e., behavior that is self-directed and deliberately results in injury or the potential for injury to oneself with evidence, either implicit or explicit, that the individual wishes to die, means to kill himself, and understands the probable consequences of his actions or potential actions) (19). Currently, SBR events are tracked in the Suicide Prevention Application Network (SPAN) database (20); however, this database does not capture events that occurred prior to October 1, 2008. It should be noted that within the ECHCS, SBR documentation commenced prior to this date. Given that the earliest TBI-4 screen in this study took place in December 2006, the text integration utilities (TIU) domain in the CDW was utilized to identify clinical notes with SBRs for each of the Veterans in our study cohort. Within the EMR, each clinical note has a title associated with it. Notes with the title of “Suicide Behavior Report” were selected. Additionally, all notes that had the key phrase “Description of event,” which is part of the SBR template used in the ECHCS EMR were also selected. Notes were pulled if they met at least one of these two criteria. Once the documents were confirmed to be SBRs, they were reviewed by two study clinicians. Using the Self-Directed Violence Classification System (19), the clinicians classified the SBR events into those that met criteria for a suicide attempt (i.e., a non-fatal self-inflicted potentially injurious behavior with any intent to die as a result of the behavior) and those that did not.

#### Statistical Analysis

Data were analyzed with SAS 9.2 or higher. Chi-squared, Fisher’s exact, or *t* tests were used as appropriate for demographic comparisons between TBI-4 positive and negative Veterans. Fisher’s exact tests were used to determine differences in the proportion of Veterans with a suicide attempt by TBI-4 status, with a significance level of 0.05. Exact Binomial 95% confidence intervals were also calculated.

## Results

Data from the CDW were used to determine Veterans’ age, sex, marital status, race/ethnicity, period of military service, and eligibility of VA service (Table 1). Characteristics of the group that screened positive on the TBI-4 differed from the TBI-4 negative group in gender (*p* = 0.001) and race/ethnicity (*p* = 0.002). Of the participants who screened positive on Question 2 of the TBI-4, 1.50% (*n* = 7) of them had a suicide attempt 1-year post-assessment compared to 0.00% (*n* = 0) of participants who were Question 2 negative (see Table 2).

**Table 1:** **Subject demographics (*n* = 1,097)**.

	TBI-4 screen		
	Positive (*n* = 468)	Negative (*n* = 629)	*p*-Value
Mean age	47.8 (±12.9)	48.2 (±13.6)	0.68
Gender			0.0001
Male	94.4% (442)	87.6% (551)	
Female	5.6% (26)	12.4% (78)	
Marital status			0.20
Married	30.8% (144)	35.5% (223)	
Divorced/separated/widowed	45.5% (213)	40.7% (256)	
Single	22.9% (107)	23.1% (145)	
Missing/unknown	0.9% (4)	0.8% (5)	
Race/ethnicity			0.002
Caucasian	76.9% (360)	68.0% (428)	
African-American	10.0% (47)	17.2% (108)	
Hispanic	0.6% (3)	0.5% (3)	
Other	1.9% (9)	3.0% (19)	
Missing	10.5% (49)	11.3% (71)	
Period of service			0.37
Vietnam Era	41.2% (193)	38.2% (240)	
Post-Vietnam	21.4% (100)	21.0% (132)	
Persian Gulf War	32.7% (153)	33.9% (213)	
Other	4.7% (22)	7.0% (44)	
Eligibility			0.69
Non-service connected	34.2% (160)	34.5% (217)	
Non-service connected, pension	5.8% (27)	4.13% (26)	
Service connected <50%	20.3% (95)	22.3% (140)	
Service connected 50–100%	39.3% (184)	38.5% (242)	
Other	0.4% (2)	0.6% (4)	

**Table 2:** **Suicide behavior reports (*n* = 1,097)**.

	Percentage of sample that had a suicide behavior report (*n*)	95% exact binomial confidence interval
Question 2 positive (*n* = 468)	1.50 (7)	0.6–3.1
Question 2 negative (*n* = 629)	0.00 (0)	0.0–0.6

*Fisher’s exact, *p* = 0.003*.

## Discussion

In a 2013 publication, Bahraini and Brenner suggested that the costs versus benefits of TBI screening should be evaluated empirically (8). That is, screening for TBI may help to identify those at risk for negative outcomes and facilitate prevention strategies. Findings from this study suggest that among those seeking mental health services, individuals who screen positive for a history of TBI are at increased risk for suicide attempt, as documented by SBRs, during the 1-year period following intake. These results support both the clinical utility and the predictive validity of the TBI-4. Moreover, when considered in the context of previous findings regarding the screening tool (1, 9), the results provide additional support for implementing the TBI-4 at the time of mental health intake.

Additional follow-up and prevention efforts among this cohort may be warranted. For example, additional and perhaps more intensive efforts aimed at engaging such individuals in treatment are likely indicated. It should be noted that for many who were evaluated, their history of TBI occurred many years prior to the mental health intake. In addition, study participants were not assessed for other comorbidities that are associated with suicidal behavior. As such, it is not clear whether the patients’ TBI is directly associated with suicidal behavior. It may have been that this history of TBI created vulnerability that was further exploited by factors that brought these individuals in for mental health treatment. This assertion is consistent with previously published work by Brenner and colleagues (8, 21) regarding the accumulation of disadvantaged risk factors among Veterans with TBI resulting in cascades of physical and psychiatric outcomes. As per Bahraini and Brenner (8) early identification of such risk factors and prevention of future accumulation may help “shift a person’s health trajectory away from chronic illness and disability.”

Since 2007, the VA has significantly increased efforts to understand Veteran suicide in an effort to reduce its occurrence (20). Data from this study along with other work in the area of TBI and suicide suggest that continued efforts to identify those with a history of TBI seeking mental health services and evaluation of potentially increased suicide risk among this cohort are warranted. Findings regarding the TBI-4 suggest that it may be a useful tool in this process. Further information regarding screening, assessment, and treatment of Veterans with TBI seeking mental health services can be found at http://www.mirecc.va.gov/visn19/tbi_toolkit/.

### Study Limitations

Several study limitations should be highlighted including the fact that only records from participants who maintained care within the ECHCS (versus those who transferred care to other VA facilities) were included, which removed 396 individuals from the initial sample. Veterans who sought care outside of ECHCS were not included in data shown here because SBRs may not have existed in the facilities where they were seeking care, as initiation of SBRs nationally did not occur until April 24, 2008. The use of SBRs at ECHCS was not fully implemented until September 2007; thus, SBRs may not have existed for the full year post-TBI-4 for a portion of our cohort. There may also have been suicide attempts that clinicians were aware of, but were not captured by SBRs leading to a possible underestimation of overall suicide attempts; however, there is no evidence suggesting that there would be a difference in reporting suicide attempts through SBRs between those with a history of TBI and those without. Finally, although our findings were significant the total number of individuals with a suicide attempt during the 1-year period was relatively small. As such, if the TBI-4 was used to identify individuals at risk for suicide attempts it would yield a high rate of false positives. However, suicide and suicidal behaviors are rare events; thus, any tool used to screen for suicidal behavior is likely to overestimate the number of individuals at risk. Considering the lack of available screening tools that can be used to identify those at increased risk for suicide, a short screener that can identify most or all individuals that are at risk for suicidal behavior, such as the TBI-4, can be very useful in clinical practice.

### Future Research

Strategies for identifying those at risk for suicide attempts and implementing evidence-based prevention interventions continues to be an area of focus within the VHA. Toward this end, future work in this area could include examination of national VHA SBR data for those with a history of TBI over a longer follow-up period. Moreover, as per Bahraini et al. (10), there is a dearth of evidence regarding suicide prevention interventions for those with a history of TBI. Further research aimed at identifying such evidence-based interventions is needed.

## Ethics Statement

This study was approved by Colorado Multiple Institution Review Board (COMIRB) and Eastern Colorado Healthcare System Research and Development Service. A waiver of consent was granted by COMIRB for the research presented in this manuscript.

## Author Contributions

LB, BH, JO-M, JH, and JF made substantial contributions to the conception and design of this research project. AS and JH were responsible for acquiring results of the TBI-4 screen from patient medical record. TH was responsible for the acquisition of data from national VA data sources. LB and BM provided clinical expertise and interpretation of notes pulled from patient medical records. TH and JF analyzed and interpreted the data for this project. AS and LB drafted this work which was then reviewed and revised by all other authors (TH, JF, BH, JO-M, BM, and JH). All authors approved the final version of the work that is being submitted.

## Disclaimer

The views expressed in this article are those of the authors and do not necessarily reflect the official policy or position of the Department of Veterans Affairs or the U.S. Government.

## Conflict of Interest Statement

The authors declare that the research was conducted in the absence of any commercial or financial relationships that could be construed as a potential conflict of interest.
